# Blockage of transient receptor potential vanilloid 4 alleviates myocardial ischemia/reperfusion injury in mice

**DOI:** 10.1038/srep42678

**Published:** 2017-02-16

**Authors:** Qian Dong, Jing Li, Qiong-feng Wu, Ning Zhao, Cheng Qian, Dan Ding, Bin-bin Wang, Lei Chen, Ke-Fang Guo, Dehao Fu, Bing Han, Yu-Hua Liao, Yi-Mei Du

**Affiliations:** 1 Research Center of Ion Channelopathy, Institute of Cardiology, Union Hospital, Tongji Medical College, Huazhong University of Science and Technology, Jiefang Avenue 1277, Wuhan, Hubei, 430022, P.R. China; 2 Department of Physiology, Nanjing Medical University, No. 140, Hanzhong Road, Nanjing, 210029, P.R. China; 3 Department of anesthesiology, Zhongshan Hospital, Fudan University, Shanghai 200032, P.R. China; 4 Department of Orhtopaedics, Union Hospital, Tongji Medical College, Huazhong University of Science and Technology, Jiefang Avenue 1277, Wuhan, Hubei, 430022, P.R. China; 5 Department of Cardiology, Xuzhou Central Hospital, Jiefang Nan Lu 199, Xuzhou, Jiangsu 221009, P.R. China

## Abstract

Transient receptor potential vanilloid 4 (TRPV4) is a Ca^2+^-permeable nonselective cation channel and can be activated during ischemia/reperfusion (I/R). This study tested whether blockade of TRPV4 can alleviate myocardial I/R injury in mice. TRPV4 expression began to increase at 1 h, reached statistically at 4 h, and peaked at 24–72 h. Treatment with the selective TRPV4 antagonist HC-067047 or TRPV4 knockout markedly ameliorated myocardial I/R injury as demonstrated by reduced infarct size, decreased troponin T levels and improved cardiac function at 24 h after reperfusion. Importantly, the therapeutic window for HC-067047 lasts for at least 12 h following reperfusion. Furthermore, treatment with HC-067047 reduced apoptosis, as evidenced by the decrease in TUNEL-positive myocytes, Bax/Bcl-2 ratio, and caspase-3 activation. Meanwhile, treatment with HC-067047 attenuated the decrease in the activation of reperfusion injury salvage kinase (RISK) pathway (phosphorylation of Akt, ERK1/2, and GSK-3β), while the activation of survival activating factor enhancement (SAFE) pathway (phosphorylation of STAT3) remained unchanged. In addition, the anti-apoptotic effects of HC-067047 were abolished by the RISK pathway inhibitors. We conclude that blockade of TRPV4 reduces apoptosis via the activation of RISK pathway, and therefore might be a promising strategy to prevent myocardial I/R injury.

Ischemia heart disease continues to be the leading cause of human disability and mortality worldwide^1^. Early coronary reperfusion is the most effective strategy to alleviate the ischemic injury; however, reperfusion itself may lead to additional myocardial injury, a phenomenon known as ischemia/reperfusion (I/R) injury^2^. The pathophysiology of myocardial I/R injury have been reviewed everywhere^2^,^3^, and involve intracellular Ca^2+^ overload and oxidase stress, which in turn initiate myocardial cell apoptosis and necrosis. However, to date no clinically approved therapy exists^3^, highlighting the need to identify the new effective targets. There is new increasing evidence that several cation-permeable transient receptor potential (TRP) channels, particularly vanilloid (TRPV) subfamily, can influence physiological systems compromised in myocardial I/R injury, and may represent potential therapeutic targets^4^.

TRPV4 channel, mainly for Ca^2+^ permeate, is widely distributed in various organs and tissues including heart and vessels^7^,^8^. It can be activated by a variety of physical and chemical stimuli, including hypotonic stimulation, cell swelling, moderate heat (>24–37 °C), and endogenous metabolites of arachidonic acid^9^,^10^. Some of these stimuli may be increased under I/R conditions. Indeed, upregulated expression of TRPV4 and enhanced TRPV4-mediated Ca^2+^ influx has been observed in the models of brain I/R^11^,^12^, while its selective antagonist HC-067047^13^ attenuated I/R induced brain injury^14^,^15^. Furthermore, sustained activation of TRPV4 dose-dependently induces apoptosis of retinal ganglion cells^16^ and neuronal death in the hippocampus^17^. Recent reports have linked excessive TRPV4 activation to heart failure^18^,^19^, suggesting it may play a key role in myocardial I/R injury.

In the present article, we firstly investigated the expression of TRPV4 in reperfused ischemic myocardium and assessed the effects of TRPV4 blockade or deletion on myocardial I/R injury in mice. Similar to our previous study^12^, we tested the dose-dependent cardioprotective effects of TRPV4 antagonist HC-067047 and further examined the efficacious time-window of HC-067047-mediated cardioprotection. Finally, we explored the molecular mechanisms and found that the protection of HC-067047 through the inhibition of apoptosis and the activation of the reperfusion injury salvage kinase (RISK) signaling pathway.

## Results

### TRPV4 increases following myocardial I/R

**Figure 1:**
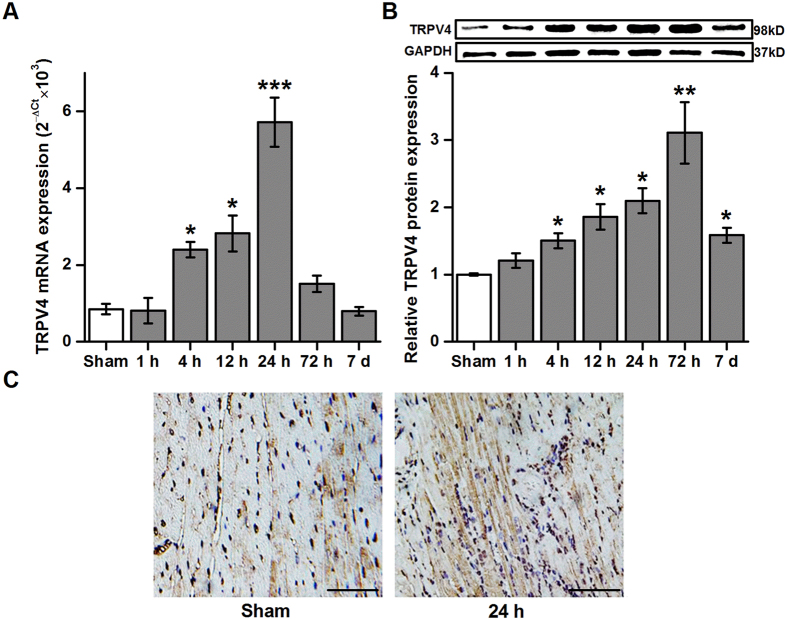
Upregulated expression of TRPV4 after myocardial I/R. Levels of TRPV4 were measured by Real-time PCR (**A**) and western blot (**B**) in the heart after 30 minutes of ischemia and reperfusion at different time. n = 6–9/group, *P < 0.05, **P < 0.01, ***P < 0.001 versus sham group. Full-length blots/gels are presented in Supplementary Fig. 5. (**C**) The expression of TRPV4 in hearts was measured at 24 h after reperfusion using immunohistochemistry. Scale bar: 50 μm.

To test the involvement of TRPV4 in myocardial I/R injury, we examined the TRPV4 expression levels in the heart at different reperfusion time points after 30 min ischemia (Supplement Fig. 1A). As shown in Fig. 1A, TRPV4 mRNA expression began to increase as early as 1 h following reperfusion, reached peak levels at 24 h and then began to decrease. Similar results were observed on TRPV4 protein abundance except that TRPV4 protein expression seems to peak at 72 h (Fig. 1B). Immunohistochemistry pictures show that more expression of TRPV4 on 24 h after I/R compared with the sham group (Fig. 1C). These results suggest that the expression of TRPV4 is up-regulated following myocardial I/R.

### TRPV4 antagonist HC-067047 reduces myocardial infarction and improves cardiac function after I/R

**Figure 2:**
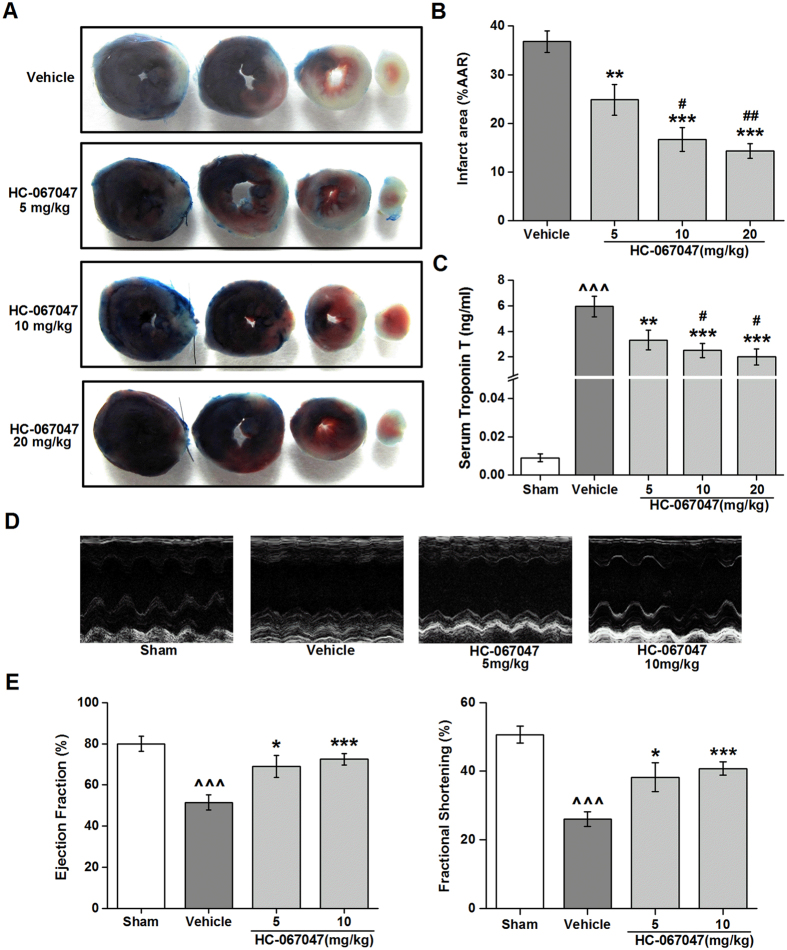
Dose-dependent effects of TRPV4 antagonist HC-067047 on myocardial I/R induced injury. **(A**) Representative images of LV slices from different doses of HC-067047 (5 mg/Kg, 10 mg/Kg, 20 mg/Kg, once/8 h) at 24 h after reperfusion as stained by Evan’s Blue and TTC. The non-ischemic area is indicated in blue, AAR in red, and the infarct area in white. (**B)** Quantification of infarct size of myocardial tissues at 24 h after reperfusion. (**C**) Serum concentration of TnT at 24 h after reperfusion. (**D)** Representative M-mode echocardiography images of the LV at 24 h after reperfusion. (**E)** Quantification of LV ejection fraction and fractional shortening at 24 h after reperfusion. n = 8/group, ^^^P < 0.001 vs sham, **P < 0.01, ***P < 0.001 vs vehicle, ^#^P < 0.05, ^##^P < 0.01 vs HC-067047 5 mg/kg.

Since myocardial I/R caused the TRPV4 up-regulation, we then sought to determine whether TRPV4 up-regulation plays an important role in myocardial I/R injury. For this, we used HC-067047, a specific inhibitor that is able to block Ca^2+^ influx mediated by TRPV4 *in vitro*^13^. We first examined the dose-dependent action of HC-067047 at 24 h after reperfusion in mice I/R model (Supplement Fig. 1B). Infarction area and area at risk (AAR) were determined by triphenyltetrazolium chloride (TTC) staining (Fig. 2A), and no significant difference in AAR/ left ventricle (LV) across all groups was found (Supplement Fig. 2A), confirming that the surgical injury was equivalent between treatment groups. However, HC-067047, given at dose ranging from 5 mg/Kg to 20 mg/Kg, reduced the infarct size in a dose-dependent manner when compared with vehicle-treated I/R mice. The level of serum troponin T (TnT) was likewise significantly decreased by HC-067046 in dose-dependent manner (Fig. 2C). Echocardiography was performed in mice injected with HC-067045 (5 and 10 mg/Kg) to evaluate cardiac function. As shown in Fig. 2D,E, mice in vehicle group exhibited marked decreases in fractional shortening (FS) and ejection fraction (EF) (P < 0.001) when compared with those in sham group. However, EF and FS were improved significant after treatment with HC-067047 as compared with vehicle group (P < 0.05 at 5 mg/Kg; P < 0.001 at 10 mg/Kg). As HC-067047 showed significant cardioprotection at a dose of 10 mg/Kg, this dose was used in the following experiment.

**Figure 3:**
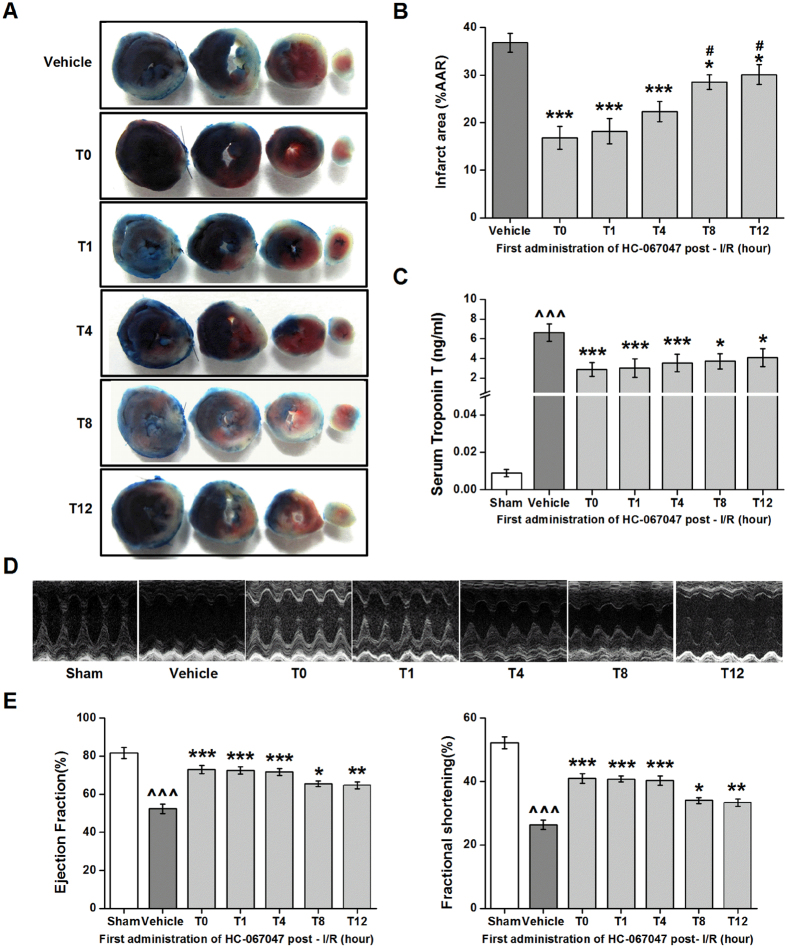
Therapeutic window of TRPV4 antagonist HC-067047. Mice were subjected to myocardial I/R and HC-067047 (10 mg/kg, once/8 h) was first given at 0 (T0), 1 (T1), 4 (T4), 8 (T8) or 12 h (T12) after the beginning of reperfusion. Representative photographs (**A**) and quantification of infarct size of myocardial tissues (**B**) at 24 h after I/R. (**C)** Serum concentration of TnT at 24 h after reperfusion. **(D)** Representative M-mode echocardiography images of the LV at 24 h after reperfusion. (**E)** Quantification of LV ejection fraction and fractional shortening at 24 h after reperfusion. n = 8/group, ^^^P < 0.001 vs sham, *P < 0.05, **P < 0.01, ***P < 0.001 vs vehicle, ^#^P < 0.05 vs T0.

Next, we determined the therapeutic window of HC-067047 on myocardial I/R injury. HC-047067 (10 mg/Kg) was first injected at 0 (T0), 1 (T1), 4 (T4), 8 (T8), or 12 (T12) h after reperfusion (Supplement Fig. 1C). Consistent with the previous study^20^, I/R in mice resulted in an average LV infarct area of 36.79 ± 2.02% when they were treated with vehicle. After administration of HC-067047 at T0, T1, T4, and T8, infarct size was reduced significantly to 16.83 ± 2.43%, 18.17 ± 2.67%, 22.33 ± 2.12%, and 28.5 ± 1.56%, respectively. Remarkably, late administration of HC-067047 at 12 h (T12) after reperfusion still had significant effects on LV infarct size 30.1 ± 2.08% (Fig. 3A,B). Likely, treatment with HC-067047 at T0, T1, T4, T8, and T12 also significantly decreased serum TnT level (Fig. 3C), but markedly increased cardiac EF and FS levels (Fig. 3D,E). These results suggest that blockage of TRPV4 reduces myocardial I/R injury with an efficacious time-window of at least 12 h.

To confirm that prolonged ischemia did not modify the protective effects of HC-067047, the duration of ischemia was extended to 1 hour and the period of reperfusion remains at 24 h (Supplement Fig. 1D). As reports in Supplement Fig. 3A,B, infarct size measured by TTC staining was significantly reduced by treatment with 10 mg/Kg HC-067047 compared with vehicle group (24 ± 2.68% vs 40 ± 1.53%, P < 0.01). The AR/LV mass ratio was unchanged (Supplement Fig. 3C). Serum TnT level was significantly reduced (Supplement Fig. 3D) and the cardiac function was markedly improved (Supplement Fig. 3E–G).

### TRPV4 knockout ameliorates myocardial I/R injury

**Figure 4:**
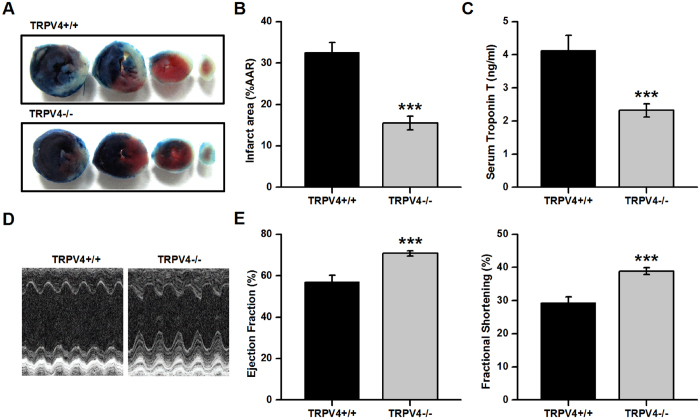
TRPV4 knockout ameliorated myocardial I/R injury. **(A)** Representative images of LV slices from TRPV4+/+ and TRPV4−/− mice at 24 h after I/R. (**B)** Quantification of infarct size of myocardium at 24 h after I/R. (**C)** Serum concentration of TnT at 24 h after reperfusion. (**D)** Representative M-mode images of the LV after sham and myocardial I/R from TRPV4+/+ and TRPV4−/− mice. (**E)** Quantification of LV ejection fraction and fractional shortening at 24 h after reperfusion. n = 12/group, ***P < 0.001 vs TRPV4+/+.

Under baseline conditions at 6–8 weeks of age, there were no significant differences between TRPV4−/− mice and their wild-type littermates (TRPV4+/+) in heart function (Supplementary Table 1, Supplement Fig. 1E). Figure 4A shows the representative photographs of IA and AAR of both TRPV4+/+ and TRPV4−/− mice after myocardial I/R. The accumulated data in Fig. 4B indicated that I/R resulted in 32.5 ± 2.4% of IA (IA/AAR) in TRPV4+/+ mice and 15.5 ± 1.6% in TRPV4−/− mice, a 52% of reduction in infarct size (P < 0.001), although the groups had comparable AAR (Supplemental Fig. 2C. Less serious injury was also manifested by lower levels of serum cTnT in TRPV4−/− mice (−44%, P < 0.001, Fig. 4C). In parallel, we observed improved cardiac function, as indicated by elevated EF (+24%) and FS (+33%), in TRPV4−/− mice after myocardial I/R compared with TRPV4+/+ (P < 0.001, Fig. 4D,E). These results further confirm that TRPV4 plays a pathogenic role in myocardial I/R injury.

### TRPV4 antagonist HC-067047 reduced cardiomyocyte apoptosis during myocardial I/R *in vivo*

**Figure 5:**
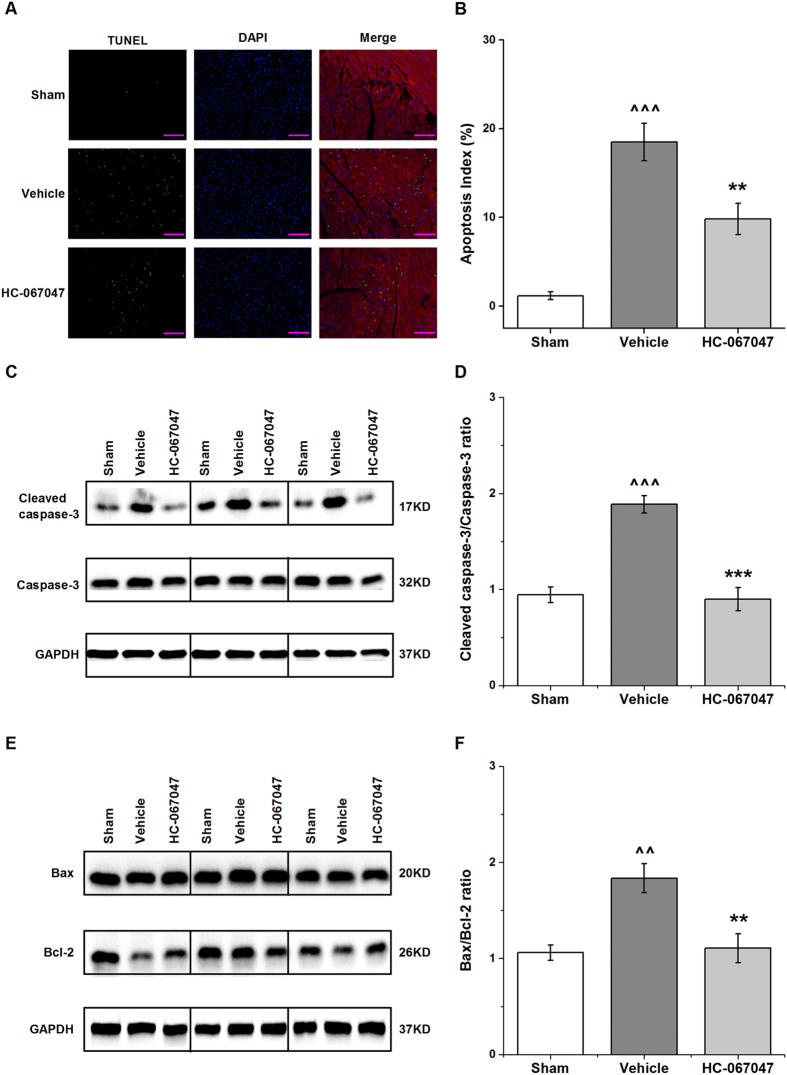
TRPV4 antagonist HC-067047 reduced cardiomyocyte apoptosis in a mouse model of myocardial I/R. The TRPV4 antagonist HC-067047 (10 mg/Kg) was intraperitoneally injected at 1 h after reperfusion. (**A)** Representative photographs of TUNEL-stained heart sections from different groups at 4 h after reperfusion. Apoptotic nuclei were identified by TUNEL staining (green), cardiomyocyte by anti-sarcomeric actin antibody (red), and total nuclei by DAPI staining (blue). Scale bar: 100 μm (**B**). Percentages of TUNEL-positive nuclei over total number of nuclei. n = 8/group, ^^^P < 0.001 vs sham, **P < 0.01 vs vehicle. (**C**) Representative photographs of cleaved caspase-3 in groups by western blot after 4 h reperfusion. Full-length blots/gels are presented in Supplementary Fig. 6. (**D)** Cleaved caspase-3 in myocardium was assessed and the values were normalized to sham, n = 6/group, ^^^P < 0.001 vs sham, ***P < 0.001 vs vehicle. (**E)** Representative photographs of Bcl-2 and Bax in groups after 4 h reperfusion. Full-length blots/gels are presented in Supplementary Fig. 7. (**F)** The results were expressed as ratio of Bax/Bcl-2. n = 6/group, ^^P < 0.01 vs sham, **P < 0.01 vs vehicle.

**Figure 6:**
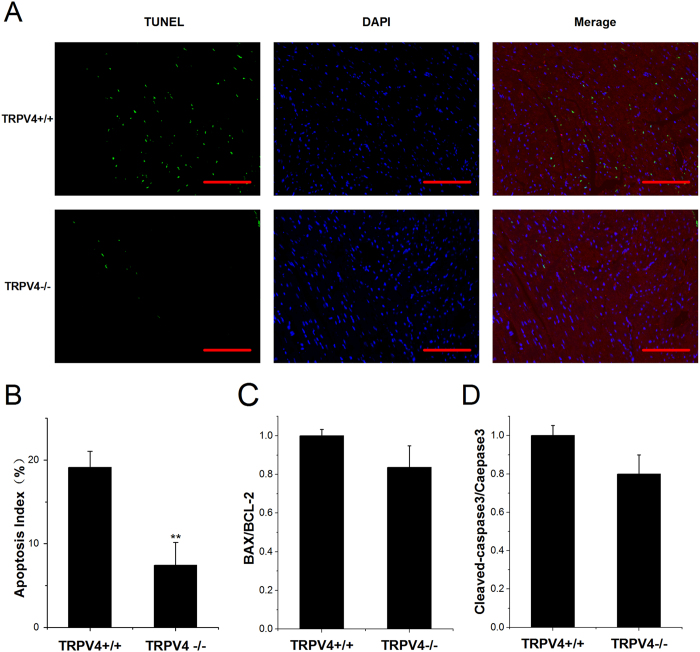
TRPV4 knockout ameliorated cardiomyocyte apoptosis in a mouse model of myocardial I/R. (**A**) Representative photographs of TUNEL-stained heart sections from different groups at 4 h after reperfusion. Apoptotic nuclei were identified by TUNEL staining (green), cardiomyocyte by anti-sarcomeric actin antibody (red), and total nuclei by DAPI staining (blue). Scale bar: 100 μm (**B)**. Percentages of TUNEL-positive nuclei over total number of nuclei. n = 3/group, **P < 0.01 vs TRPV4+/+. (**C**) Bcl-2 and Bax in myocardium of TRPV4+/+ and TRPV4−/− mice after 4 h reperfusion. Results were expressed as ratio of Bax/Bcl-2. n = 3/group. (**D**) Cleaved caspase-3 in myocardium of TRPV4+/+ and TRPV4−/− mice after 4 h reperfusion was assessed and the values were normalized to TRPV4+/+ mice, n = 3/group.

Previous studies have demonstrated that up-regulation of TRPV4 can effectively induce apoptosis, suggesting a possible link between TRPV4 and apoptosis pathway^13^,^16^,^21^. To determine the underlying mechanism for the reduced myocardial injury by HC-067047 administration, we investigated the effects of HC-067047 on myocardial apoptosis induced by I/R. TUNEL and cardiomyocyte-specific sarcomeric actinin double staining were performed on the LV sections from a sham animal, and I/R animals after treatment with vehicle and HC-067047 at 4 h after myocardial I/R. Representative images from each group were shown in Fig. 5A and Supplement Fig. 4A. Quantitative analysis revealed that HC-067047 treatment significant reduced the frequencies of TUNEL-positive myocytes compared with vehicle group (Fig. 5B). Consistent with TUNEL staining, the induction in cleaved caspase-3 expression in response to I/R in ischemic heart was significantly suppressed by HC-067047 treatment (Fig. 5C,D). Furthermore, we examined the effects of TRPV4 on expression of proapoptotic protein Bax and antiapoptotic protein Bcl-2 using western blot (Fig. 5E,F). The Bax/Bcl-2 ratio was significantly increased in heart subjected to I/R. HC-067047 treatment decreased Bax/Bcl-2 ratio. Less TUNEL index was observed in TRPV4−/− mice at 4 h after I/R, as compared to TRPV4+/+ controls (Fig. 6A,B and Supplement Fig. 4B). However, Bax/Bcl2 and cleaved caspase-3 comparison between the TRPV4−/− and TRPV4+/+ group did not reach significance (Fig. 6C,D). This result suggested that the anti-apoptosis mechanism of deletion TRPV4 gene may be different from that of TRPV4 antagonist HC-067047, which require further studies. Indeed, the caspase-independent apoptosis pathway, including the release of apoptosis inducing factor and endonuclease G from mitochondria, plays the important role in I/R injury and is likely to be related with the cardioprotective effects^22^,^23^.

### TRPV4 antagonist HC-067047 obviously increased phosphorylation of Akt, ERK1/2 and GSK-3β in the myocardium after I/R

**Figure 7:**
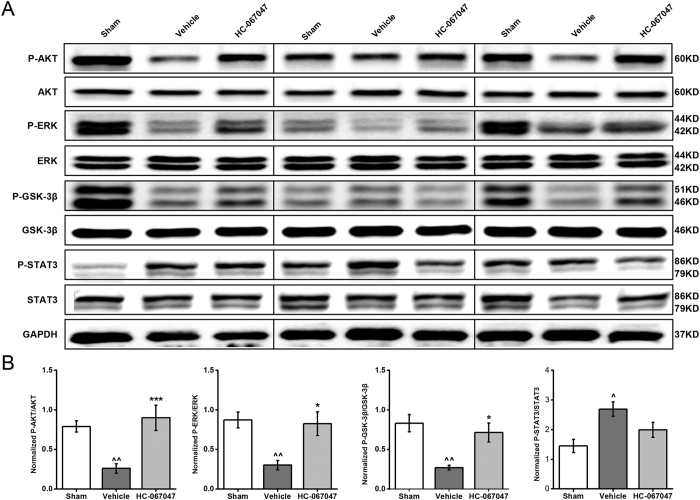
Effects of TRPV4 antagonist on the cardiac expression of activated RISK (P-Akt, P-ERK, P-GSK-3β) and SAFE (P-STAT3) pathways at 4 h after myocardial I/R in mice. The TRPV4 antagonist HC-067047 (10 mg/Kg) was intraperitoneally injected at 1 h after reperfusion. Representative Western blot images are shown in A, quantification in B. n = 6/group, ^P < 0.05, ^^^P < 0.001 vs sham, ***P < 0.01 vs vehicle. Full-length blots/gels are presented in Supplementary Figs 8–11.

We further investigated the potential mechanisms responsible for the cardioprotective effects of TRPV4 inhibition. For these studies, we focused on components of the RISK pathway, a signaling cascade involving prosurvival kinases, including PI3K/Akt, ERK1/2, and the downstream target GSK-3β. The concept for the RISK pathway is based on the evidence that activation of certain kinases exerts anti-apoptotic effects^24^. Therefore, it has been postulated that targeting these kinases at the time of reperfusion with pharmacological agents would protect the myocardium^25^. For these studies, mice were again subjected to 30 minutes of myocardial ischemia and 4 h of reperfusion. Western blot analysis of left ventricle homogenates from sham, vehicle HC-067047 treated mice revealed that I/R injury significantly decreased the levels of phosphorylated Akt, ERK1/2 and GSK-3β (P < 0.001) in the vehicle-treated mice, compared with sham group. However, HC-067047 treatment markedly increased the levels of phosphorylated Akt (P < 0.001), ERK1/2 (P < 0.05) and GSK-3β (P < 0.05), as compared with the vehicle group (Fig. 7). We also examined whether the survival activating factor enhancement (SAFE) pathway^26^, which involves activation of TNF-α and the transcription factor STAT3, involved in TRPV4 blockade mediated cardiac protection. As shown in Fig. 7, I/R induced STAT3 activation and HC-067047 did not alter I/R-induced STAT3 phosphorylation. Taken together, these results suggest that the protective role of HC-067047 in myocardial I /R injury is related to RISK pathway by the activation of the PI3K/Akt, ERK1/2 and GSK-3β, but not the SAFE pathway. In addition, the levels of phosphorylated Akt and ERK1/2 at 4 h after I/R were determined in TRPV4−/− mice and TRPV4+/+ controls and showed similar in both groups (P > 0.05, data not shown). This result suggested that the cardioprotective effects of deletion TRPV4 gene may be through other molecular mechanisms instead of RISK signaling, which require further studies.

### Pharmacological inhibition of RISK pathways abolished the cardioprotective effect of TRPV4 antagonist HC-067047

**Figure 8:**
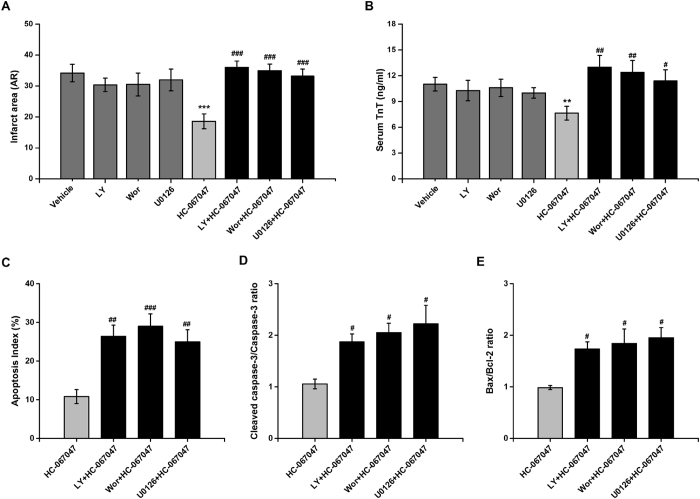
RISK signaling contributes to the protective of TRPV4 antagonist HC-067047 on myocardial IR injury. Mice were treated with HC-067047 at 1 h after reperfusion. Inhibitors of RISK pathway, LY294002 (LY) wortmannin (Wor), U0126 were administrated 60 min before ischemia. (**A)** Quantification of infarct size of myocardium at 4 h after I/R. (**B**) Serum concentration of TnT at 4 h after reperfusion. (**C)** Percentages of TUNEL-positive nuclei over total number of nuclei. (**D)** Cleaved caspase-3 to caspase-3 ratio. (**E)** Bax to Bcl-2 ratio at 4 h after I/R. n = 6–8/group, **P < 0.01, ***P < 0.001 vs vehicle, ^#^P < 0.05, ^##^P < 0.01, ^###^P < 0.001 vs HC-067047.

To further examine the increased activation of the RISK pathway involved in the protection of the myocardium from I/R injury in HC-067047 post-treated mice, we administered the PI3K inhibitor Wortmannin and LY294002, and ERK1/2 inhibitor to mice to determine whether the cardioprotective effect of HC-067047 can be abolished by these inhibitors (Supplement Fig. 1F). The results demonstrated that HC-067047 administrated at 1 h after myocardial reperfusion, remarkably reduced the infarct size from 34.2 ± 2.85% in vehicle to 18.6 ± 2.4% in HC-067047 (P < 0.001; Fig. 8A). Simultaneously administration of LY294002, Wortmannin, or U0126 completely abolished the infarct size reduction observed in the HC-067047-treated hearts. Additionally, none of these inhibitors had significantly effect on infarct size when given alone (Fig. 8A). The AAR of myocardial infarction was comparable among the treatment groups (Supplemental Fig. 2D). Consistent with the above results, these inhibitors also abolished serum TnT level reduction effect of HC-067047 (Fig. 8B). Moreover, all these inhibitors abolished the anti-apoptotic effects of HC-067047, as evidenced by the decrease in the apoptotic index, the cleaved caspase-3 expression and the Bax/Bcl-2 ratio (Fig. 8C–E). These results further confirmed the protective effects of TRPV4 blocker HC-067047 were mediated by RISK pathway.

## Discussion

The present study is the first to reveal a critical role of TRPV4 in myocardial I/R injury. TRPV4 mRNA and protein expression is significant upregulated after myocardial I/R. In addition, inhibition or delete TRPV4 ameliorated myocardial I/R injury as demonstrated by reduced infarct size, decreased TnT and improved cardiac function. More importantly, TRPV4 antagonist HC-067047 dose-dependently protects the heart against I/R injury when given even 12 h after reperfusion. We also demonstrate that this effect relies on reduction of myocardial apoptosis via the activation of RISK pathway including PI3K-Akt, ERK1/2, and GSK-3β. These data highlight the potential therapeutic role of TRPV4 in cardioprotection in response to acute myocardial I/R injury.

TRPV4 is wildly expressed in the cardiovascular system, particularly in heart^7^ as well as isolated cardiomyocytes^27^,^28^,^29^. However, there are no data demonstrating the role of TRPV4 in heart under either physiological or pathophysiological conditions, such as myocardial I/R. We found that the levels of TRPV4 mRNA and protein increased in a time dependent manner after reperfusion. Although the level of TRPV4 mRNA increased significantly at 24 h after reperfusion, the level of TRPV4 protein did increase at 72 h, which is likely due to the synthesis or degradation of mRNA and protein inconsistent speed. A similar finding of the increase TRPV4 expression has been described in neuronal injury after cerebral I/R^11^,^12^. We further assessed the effects of TRPV4 blockades on infarct size, TnT and cardiac function using both TRPV4 antagonist HC-067047 and TRPV4 knockout mice. Our results demonstrated TRPV4 blockades significant reduced the myocardial I/R injury, suggesting that TRPV4 involved in myocardial I/R injury. In addition, we examined the dose-dependent effects of TRPV4 antagonist HC-067047 and found that the effective dose ranged from 5 to 20 mg/Kg in mice. Specifically, HC-067047 at 10 mg/Kg was injected beginning from 0 to 12 h after perfusion to determine its therapeutic time-window. It was found that HC-067047 produced significant reduction in infarct size, decreased the TnT and improved heart function when first administrated was initiated within 12 h after reperfusion. Therefore, TRPV4 antagonist HC-067047 dose-dependently protects heart against I/R injury with an efficacious time-window of at least 12 h, which consisted with the previous report using the mode of brain I/R injury^15^. Based on our knowledge, the time window of protection afforded by postconditioning with TRPV4 antagonist HC-067047 is much larger than previous reports^30^, supporting the therapeutic potential of TRPV4 blockade for the treatment of myocardial I/R injury.

Apoptosis has been proposed to play a significant role in injury induced by myocardial I/R^31^. It is mediated by caspase 3, a main death executing protein. Moreover, Bcl-2 family consists of pro- and anti-apoptotic members. The equilibrium between Bax and Bcl-2 determines apoptosis induction^32^. Excess Ca^2+^ entry mediated via TRPV4 has been reported to trigger apoptosis in several cells^16^,^17^,^21^,^33^. For example, application of TRPV4 agonist promoted the dose-dependent apoptosis of retinal ganglion cells^16^ and neurons in the hippocampus^17^. Here, we found that myocardial I/R induced severe apoptosis, as confirmed by the increase of TUNEL positive cardiomyocytes, Bax/Bcl-2 ratio as well as cleaved caspase-3. However, TRPV4 antagonist HC-067047 treatment significant reduced cardiomyocyte apoptosis. Therefore, activation of TRPV4 may trigger cardiomyocyte apoptosis in myocardial I/R injury.

RISK (Akt/ERK/GSK-3β) and SAFE (JNK/STAT3) signaling cascades are critical signaling pathways involved in antiapoptotic activities in I/R injury^26^, and previous in brain I/R injury data suggest that blockade TRPV4 can activate them^17^. Our results demonstrate that blockade of TRPV4 with HC-067047 induced significantly greater phosphorylation of Akt, ERK1/2 and GSK-3β at 24 h after reperfusion compared to vehicle group, but with no effects on phosphorylation of STAT3. Therefore, in a murine model of myocardial I/R, HC-067047 activates RISK pathway. Consistent with this, treatment with HC-067047 caused a decreased in apoptosis as shown by a reduction in TUNEL positive cells, the Bax/Bcl-2 ratio and cleaved caspase-3. Importantly, the anti-apoptotic effects of treatment with TRPV4 antagonist HC-067047 were abolished by the specific pharmacological blockers of the RISK pathway (LY294002, Wortmannin and U0126), which indicate that the cardioprotective actions of blockade TRPV4 are mediated by RISK pathway.

In conclusion, we clearly demonstrate that activation of TRPV4 is upregulated during myocardial in I/R and inhibition or delete TRPV4 attenuates the myocardial I/R injury. Blockade TRPV4 with HC-067047 reduced infarct size, decreased TnT and improved the heart function with a wide therapeutic window. These cardioprotective effects of treatment with TRPV4 antagonist HC-067047 in murine model of myocardial I/R are mediated by activation of RISK pathway, resulting in a reduction of myocardial apoptosis. Our findings suggest that TRPV4 may be a promising target for treatment of myocardial I/R injury and warrant further investigation.

## Methods

### Animals

Male C57BL/6 mice of 6–8 weeks were purchased from Vital River Laboratories, Beijing, China. TRPV4 knockout mice with a C57BL/6 background were kindly provided by Dr. Atsuko Mizuno (Jichi Medical University)^34^. The mice housed in a temperature and humidity controlled room with a 12/12-h light-dark cycle, and fed with standard laboratory animal chow with free access to tap water. All experiments were performed in adherence with the National Institutes of Health Guidelines on the Use of Laboratory Animals and were approved by the Tongji Medical School, Huazhong University of Science and Technology Committee on Animal Care. All efforts were made to minimize animal suffering and to reduce the number of animals used.

### *In vivo* myocardial I/R protocol

The surgical procedures of I/R were performed as previously described^35^. Briefly, mice (22–23 g) were anesthetized by intraperitoneal injection of pentobarbital sodium (50 mg/kg), and the heart was exposed through a left thoracotomy at the forth intercostal space. The slipknot was tied around the left anterior descending coronary artery (LAD) 2–3 mm from its origin under a surgical microscope. Successful LAD occlusion was confirmed by ST-segment elevation on ECG (BL-420F, Taimeng instrument, Chengdu, China) and the appearance of myocardial pallor. The slipknot was released after 30 min of ischemia to allow reperfusion. Sham-operated (sham) animals were subjected to the same surgical procedures except that the slipknot was not tied.

### Experimental groups and drug administration

For the measurement of TRPV4 expression patterns, C57BL/6 mice were subject to sham or 30 minutes ischemia followed by 1, 4, 12, 24, 72 hours and 7 days reperfusion

To evaluate a cardioprotective effect of TRPV4 inhibition, C57BL/6 mice were treated with the specific TRPV4 antagonist HC-067047 (Sigma) after I/R. HC-067047 was dissolved with 1% dimethyl sulfoxide (DMSO) in normal saline and administrated by an intraperitoneal injection every 8 h^13^,^17^,^36^. For dose-response experiments, mice were treated with 3 different doses of HC-067047 (5, 10, and 20 mg/kg) at the beginning of reperfusion. For therapeutic time windows experiments, HC-067047 (10 mg/kg) was firstly injected 0 h (T0), 1 h (T1), 4 h (T4), 8 h (T8), and 12 h (T12) after reperfusion. In addition, TRPV4−/− mice were underwent I/R surgical procedure, while TRPV4+/+ mice were also subject to I/R as wild-type controls. Twenty-four hours later, all the mice were anesthetized with 1.5% isoflurane, and echocardiographic analysis of cardiac function was recorded. Thereafter, all animals were sacrificed and blood samples were collected in dry test tubes without coagulant to get serum for TnT levels assay (Roche Diagnostics GmbH, Mannheim, Germany). Heart tissues were harvested immediately for determination of myocardical infarct size.

To investigate the mechanisms underlying the cardioprotective effects of HC-067047 on I/R, mice were assigned randomly into three groups: sham, vehicle, and 10 mg/kg HC-067047 groups. At 4 hours post reperfusion, mice were used to analyze apoptosis (TUNEL staining, cleaved caspase-3, caspase-3, Bcl-2 and Bax protein expression) and the expression of RISK signaling molecules (P-Akt/Akt, P-ERK1/2/ERK, P-GSK-3β/ GSK-3β) and SAFE signaling molecules (P-STAT3/STAT3)^26^.

To further determine the role of RISK signaling in HC-067047 induced cardioprotective effects, mice were administered with LY294002 (Selleckchem, an inhibitor of PI3K, 40 mg/kg, i.p.), wortmannin (Selleckchem, an inhibitor of PI3K, 1 mg/kg, i.p.), or U0126 (Selleckchem, an inhibitor of ERK1/2, 1 mg/kg, i.p.), at 1 h before the mice underwent I/R. The dose and timing of LY294002, wortmannin, and U0126 were selected according to previous studies^37^,^38^. HC-067047(10 mg/Kg) was administered at 1 h after reperfusion. Hearts were then removed at 4 h after reperfusion for further analysis.

### Determination of myocardical infarct size

Mice were briefly re-anesthetized at the end of the reperfusion period, and the LAD was re-ligated and 1 ml of 1% Evans Blue dye was infused into the aorta to delineate the AAR. The LV was isolated and cut into 1-mm-thick transverse slices. In order to differentiate infarcted from viable tissue, slices were incubated in 1% TTC in phosphate buffer at pH 7.4 at 37 °C for 10 min, then they were fixed with 10% formaldehyde for 24 h and photos were taken. Regions negative for Evans Blue staining (AAR, red and white) and negative for TTC (infarct area, white) were calculated by a blinded observer using the computer-assisted planimetry function in ImageJ 6.0 (NIH, Bethesda, MD). The myocardial infarct size was expressed as a percentage of infarct area over total AAR.

### Echocardiographic analysis of cardiac function

A Vevo 2100 high-resolution microimaging system with a 30 MHz transducer was used (Visualsonic, Toronto, Ontario, Canada). Mice were anesthetized with 1.5% isoflurane and two-dimensional echocardiographic views of the mid-ventricular short axis and parasternal long axes were obtained. M-mode images were used to measure LV and LV EF and FS, which were acquired by a technician who was blinded to the treatment groups. Data analysis was performed using the VisualSonics data analysis suite.

### Immunohistochemistry

The heart tissues were fixed with 4% formalin, embedded in paraffin, and sectioned into 5-μm thick slices. The sections were stained with primary antibodies against TRPV4 (#ACC-034, Alomone labs, Israel) followed by incubation with biotin-conjugated secondary antibodies, and then treated with avidin-perioxidase. The reaction was developed using the DAB substrate kit (Biossci, Wuhan, China), and the sections were then counterstained with haematoxylin-eosin. Staining was examined using light microscopy (Advanced microscopy group, Bothell, WA, USA).

### Real-Time PCR

Real-time PCR was performed as previously described^39^,^40^. The total LV RNA was isolated using TRIzol (Invitrogen, Carlsbad, CA, USA). Melting curves established the purity of the amplified band after 40 cycles of 30 seconds at 94 °C, 30 seconds at 57 °C, and 30 seconds at 72 °C. Amplification reactions were performed in duplicate. The results of the real-time PCR data were represented as Ct values, where Ct was defined as the PCR threshold cycle at which amplified product was first detected. Gene expression levels relative to GAPDH were determined using the 2^−ΔCt^ method^41^. The primers used as followed: TRPV4, 5′-CGT CCA AAC CTG CGA ATG AAG TTC-3′ (forward) and 5′-CCT CCA TCT CTT GTT GTC ACT GG-3′ (reversed); and GAPDH, 5′-CAT GAG AAG TAT GAC AAC AGC CT-3′ (forward) and 5′-AGT CCT TCC ACG ATA CCA AAG T-3′ (reverse).

### Western blot

Western blotting was conducted according to standard protocols as previously described^42^. Protein-extracts (20 μg) of snap-frozen whole LV tissues were run on a 10% or 12% SDS-PAGE gel following by blotting to a nitrocellulose membrane. Western blot band quantifications were performed with Image Lab software (Bio-Rad, Richmond, CA, USA), and the relative values were expressed relative to GAPDH signals. Antibodies against the following proteins were used: TRPV4 (#ACC-034, Alomone labs), Bax (#2772,Cell Signaling), Bcl-2 (#2870, Cell Signaling), Caspase-3 (#19677, Proteintech), GAPDH (Guge), Akt (#4691,Cell Signaling), P-Akt (#4060, Cell Signaling), ERK (#4695, Cell Signaling), P-ERK (#4370, Cell Signaling), GSK-3β (#12456, Cell Signaling), P- GSK-3β (#9331, Cell Signaling). STAT3 (#9132, Cell Signaling) and P-STAT3 (#9131, Cell Signaling).

### TUNEL assay

Myocardial apoptosis was assessed by TUNEL staining^20^. The paraffin-embedded LV tissue was cut into sections 4 μm thick. Tunel staining was performed using the *in situ* Cell Death Detection kit (#11684817910, Roche Diagnostics) for apoptotic cell nuclei. Following this, sections were co-stained with anti-sarcomeric actinin antibody (Sigma-Aldrich, St. Louis, MO, USA) to specifically mark cardiomyocytes. TRITC goat anti-mouse antibody was applied as secondary antibody. Total nuclei were stained with DAPI. All sections were photographed at 20 × objective with an Olympus BX-51 epifluorescence microscope. Total nuclei (blue) and the TUNEL-positive nuclei (green) were counted by ImageJ 6.0. Apoptotic index was calculated automatically as a percentage of TUNEL-positive nuclei over total number of DAPI -stained nuclei. Results from a total of 40 fields per heart were averaged and counted as one sample. All of these assays were performed in a blinded manner.

### Statistical analysis

Results are reported as mean ± standard error of the mean (SEM) for at least three independent experiments. Two-tailed *t* tests or one-way ANOVA followed by Bonferroni’s post-hoc test were performed to analyze differences with group comparison. Values of P < 0.05 were considered statistically significant.

## Additional Information

**How to cite this article**: Dong, Q. *et al*. Blockage of transient receptor potential vanilloid 4 alleviates myocardial ischemia/reperfusion injury in mice. *Sci. Rep.* **7**, 42678; doi: 10.1038/srep42678 (2017).

**Publisher's note:** Springer Nature remains neutral with regard to jurisdictional claims in published maps and institutional affiliations.

## Supplementary Material

Supplementary Material
