# Supplementary material for: Blockage of transient receptor potential vanilloid 4 alleviates myocardial ischemia/reperfusion injury in mice
Source: Sci Rep. 2017 Feb 16;7:42678. doi: 10.1038/srep42678 (PMC5311718; doi:10.1038/srep42678)
Supplement: Supplementary Material [file srep42678-s1.doc]

**Blockage of** **transient receptor potential vanilloid 4 alleviates** **myocardial ischemia/reperfusion injury** **in mice**

**Qian Dong^1+^, Jing Li^1+^, Qiong-feng Wu^1+^,** **Ning Zhao^1^, Cheng Qian^1^, Dan Ding^1^, Bin-bin Wang^1^, Lei Chen^2^, Ke-Fang Guo^3^,** **Dehao Fu^4^, Bing Han^5^,Yu-Hua Liao^1^ , Yi-Mei Du^1^***

^1^Research Center of Ion Channelopathy, Institute of Cardiology, Union Hospital, Tongji Medical College, Huazhong University of Science and Technology, Jiefang Avenue 1277, Wuhan, Hubei, 430022, P.R. China

^2^Department of Physiology, Nanjing Medical University, No. 140, Hanzhong Road, Nanjing, 210029, P.R. China

^3^Department of anesthesiology, Zhongshan Hospital, Fudan University, Shanghai 200032, P.R. China

^4^Department of Orhtopaedics, Union Hospital, Tongji Medical College, Huazhong University of Science and Technology, Jiefang Avenue 1277, Wuhan, Hubei, 430022, P.R. China

^5^Department of Cardiology, Xuzhou Central Hospital, Jiefang Nan Lu 199, Xuzhou, Jiangsu 221009, P.R. China.

**^*^corresponding author:** Yi-Mei Du, E-mail: [yimeidu@mail.hust.edu.cn](mailto:yimeidu@mail.hust.edu.cn)

**^+^** These authors contribute equally to this work.

**Supplementary Table 1: Echocardiographic analysis of TRPV4*^–/–^* mice at baseline.**

| **Group** | **TRPV4+/+** | **TRPV4–/–** |
| --- | --- | --- |
| Age (week) | 6-8 | 6-8 |
| N | 6 | 6 |
| LVEDD (mm) | 3.53±0.06 | 3.39±0.08 |
| LVESD (mm) | 2.0±0.04 | 1.92±0.06 |
| EF | 75.18±0.49 | 75.63±0.82 |
| FS | 43.23±0.4 | 43.42±0.66 |
| HR (beat/min) | 482±20 | 484±17 |

**Supplementary Figure 1. Experimental protocols and genotyping of TRPV4 -/- mice.** Mice underwent a surgical protocol of myocardial ischemia/reperfusion (IR).


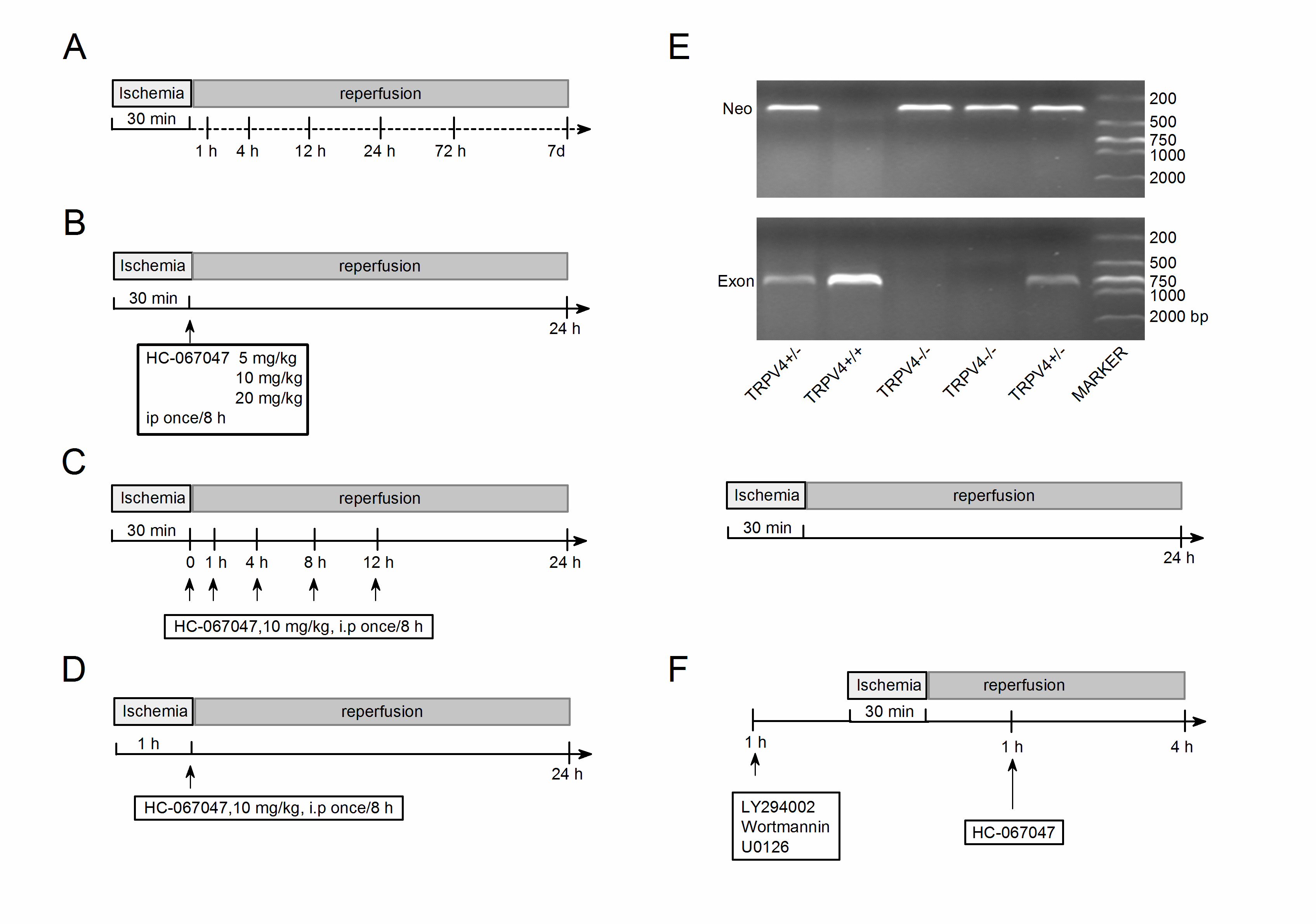


**Supplementary Figure 2. Area at risk (% left ventricular area) in different groups of mice.** There were no differences in AAR (%LV) among groups.


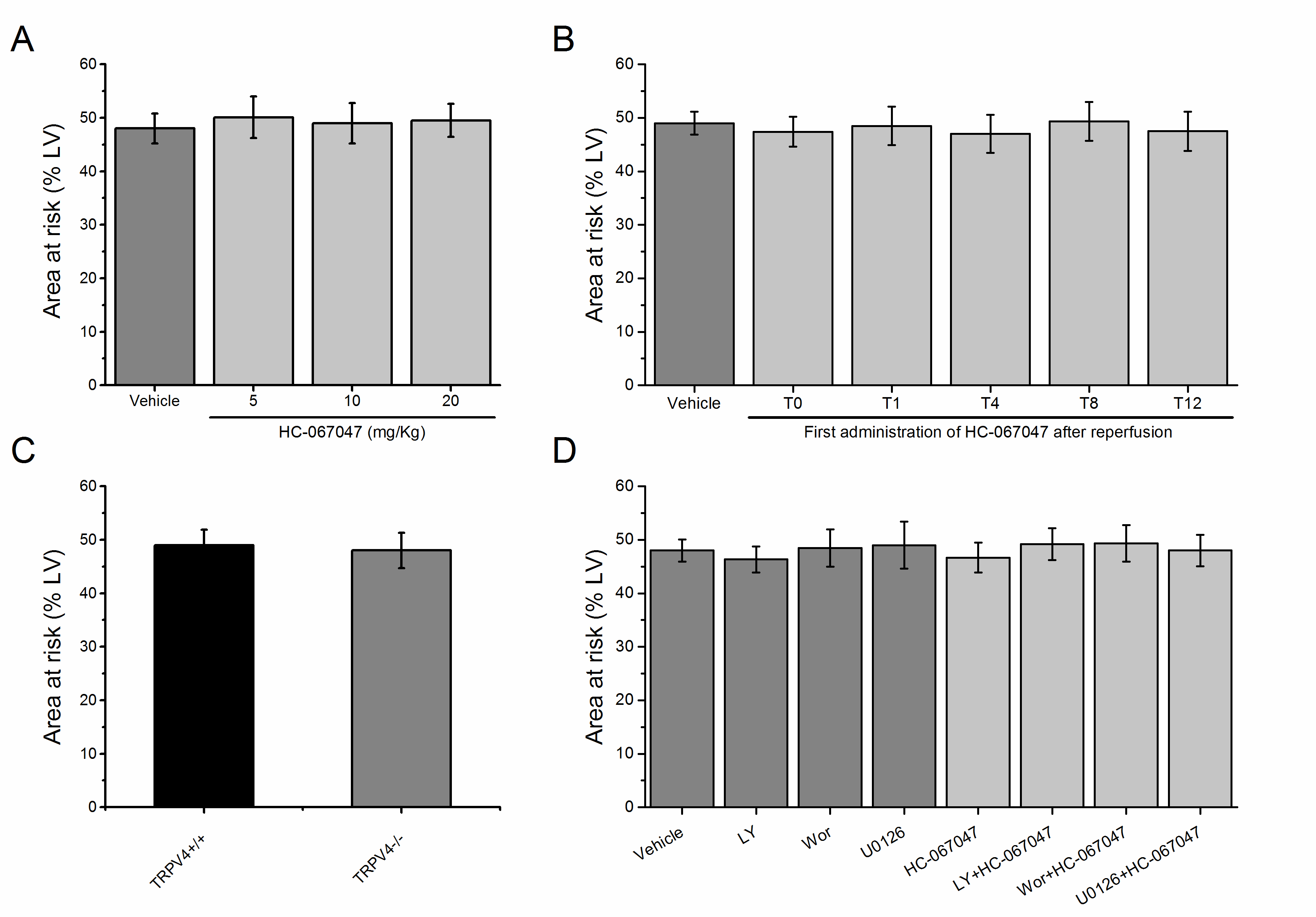


**Supplementary Figure 3. The TRPV4 antagonist HC-067047 reduce myocardial damage in mice subjected to 60 min ischemia followed by 24 h reperfusion.** HC-067047 (10 mg/kg once/8 h) was first given at the beginning of reperfusion. A. Representative photographs (A), quantification of infarct size of myocardial tissues (B) and area at risk (% left ventricular area) (C) at 24 h after reperfusion. D. Serum concentration of TnT at 24 h after reperfusion. E. Representative M-mode echocardiography images of the LV at 24h after reperfusion. F. Quantification of LV ejection fraction and fractional shortening at 24 h after reperfusion, n=6/group, ^^^P<0.001vs sham, *P<0.05， ** P<0.01, ***P<0.001 vs vehicle.


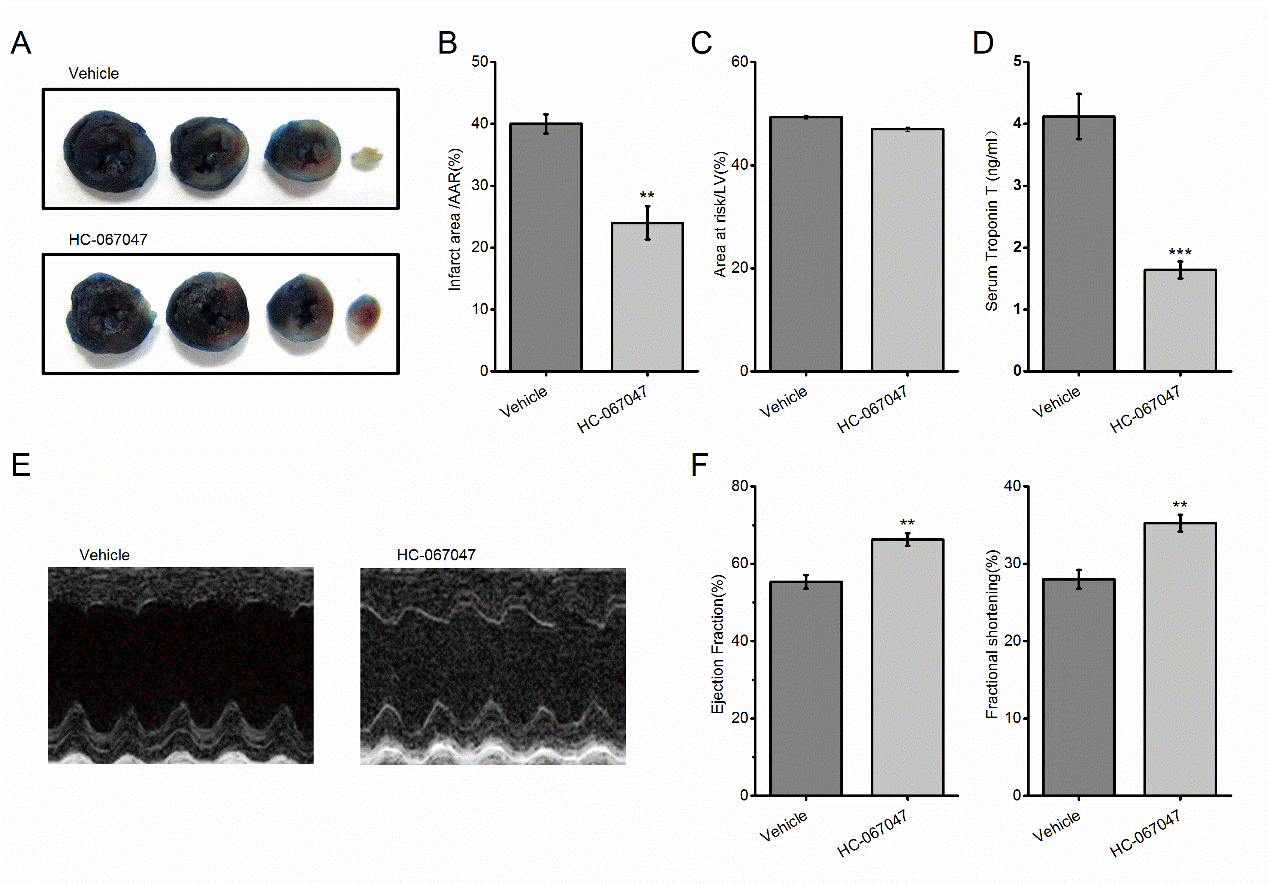


**Supplementary Figure 4. Representative photographs of TUNEL-stained heart sections in greater magnificatio**n. Apoptotic nuclei were identified by TUNEL staining (green), cardiomyocyte by anti-sarcomeric actin antibody (red), and total nuclei by DAPI staining (blue). Scale bar: 50 μm. **A.** Representative photographs of TUNEL-stained heart sections from different groups at 4 h after reperfusion.TRPV4 antagonist HC-067047 (10 mg/Kg) was intraperitoneally injected at 1 h after reperfusion. **B.** Representative photographs of TUNEL-stained heart sections from TRPV4+/+ and TRPV4-/- mice at 4 h after reperfusion.


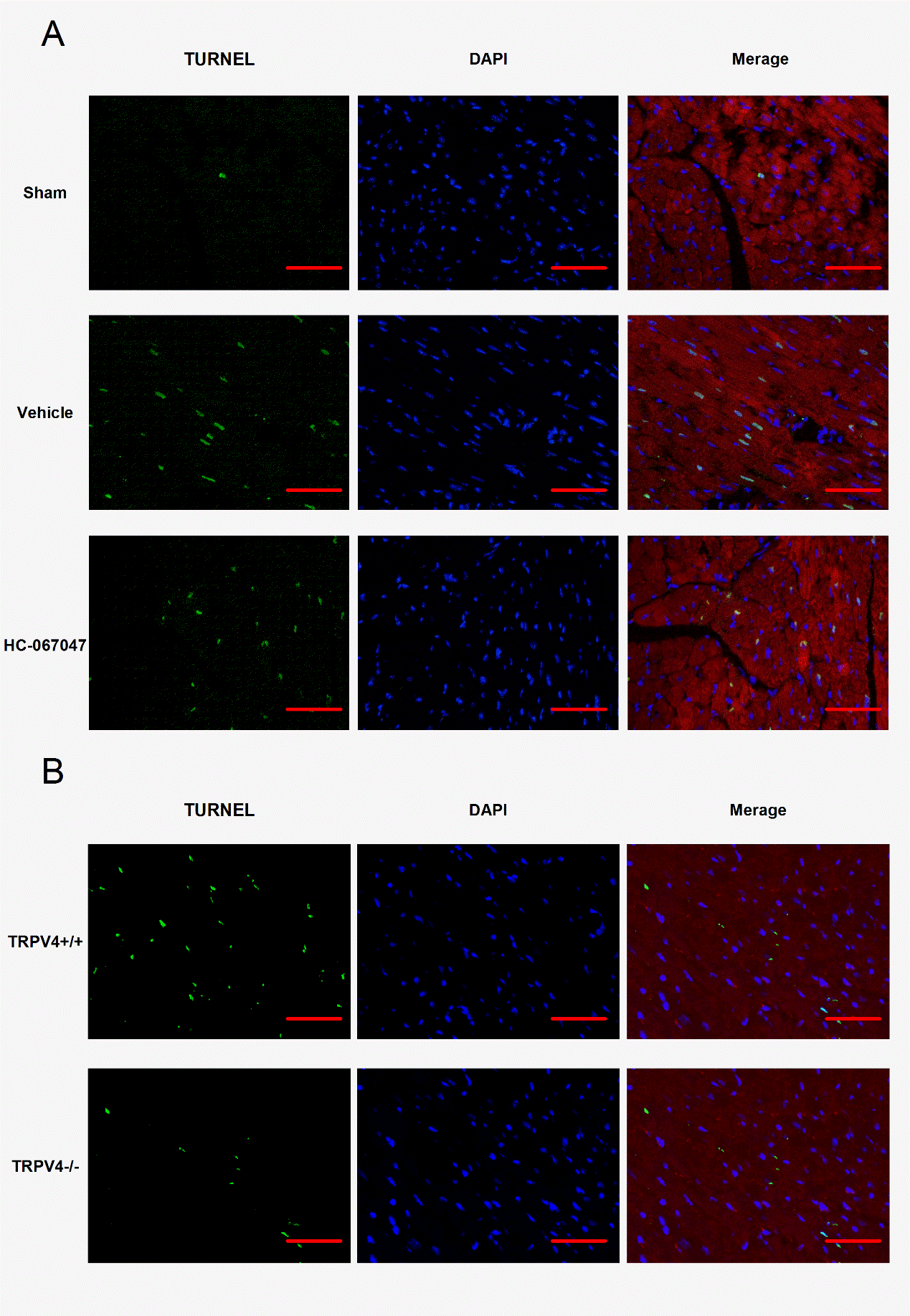


**Supplementary Figure 5 . The full-length blots/gels is the display of cropped gels and blots from Figure 1B.**


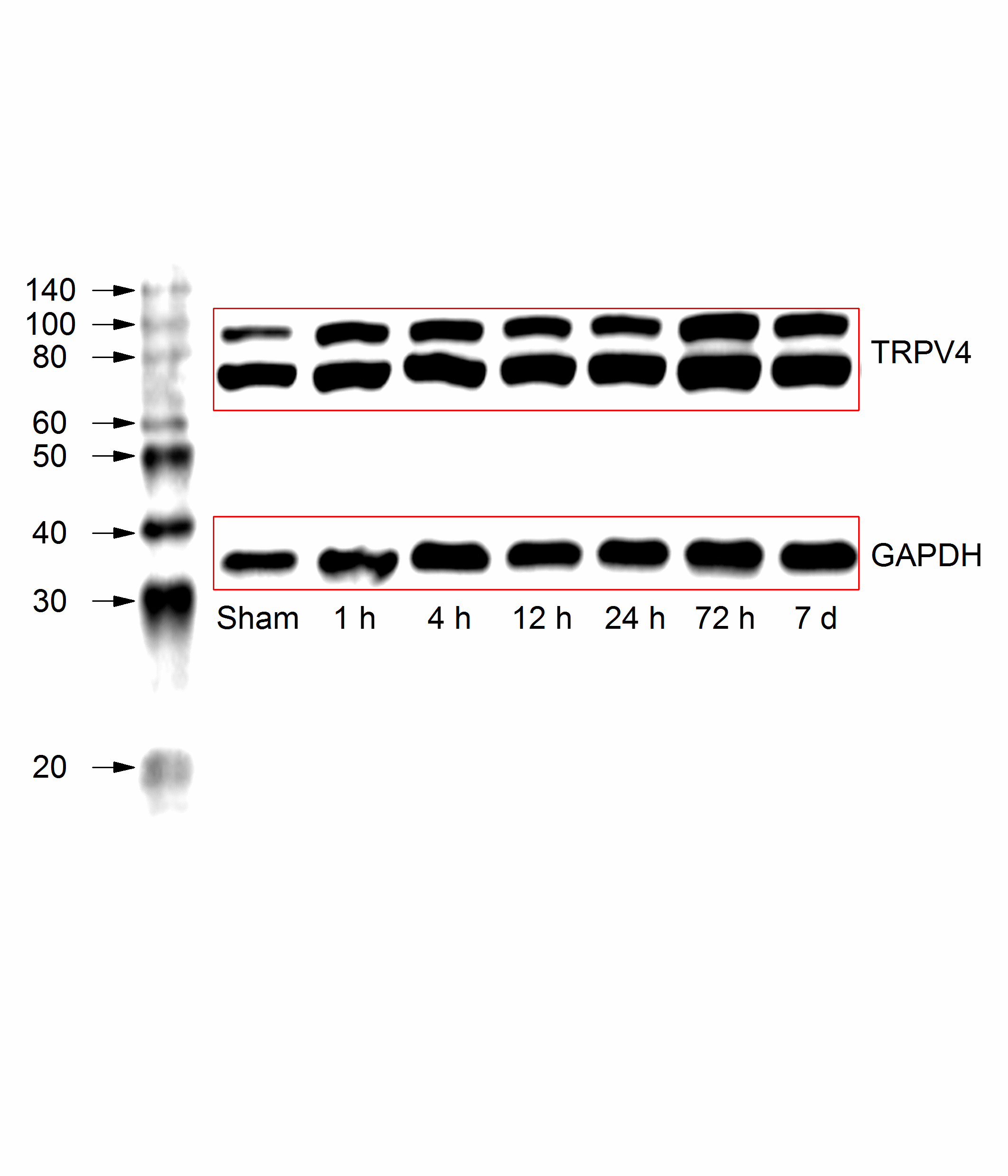


**Supplementary Figure 6 . The full-length blots/gels is the display of cropped gels and blots from Figure 5C.**

**
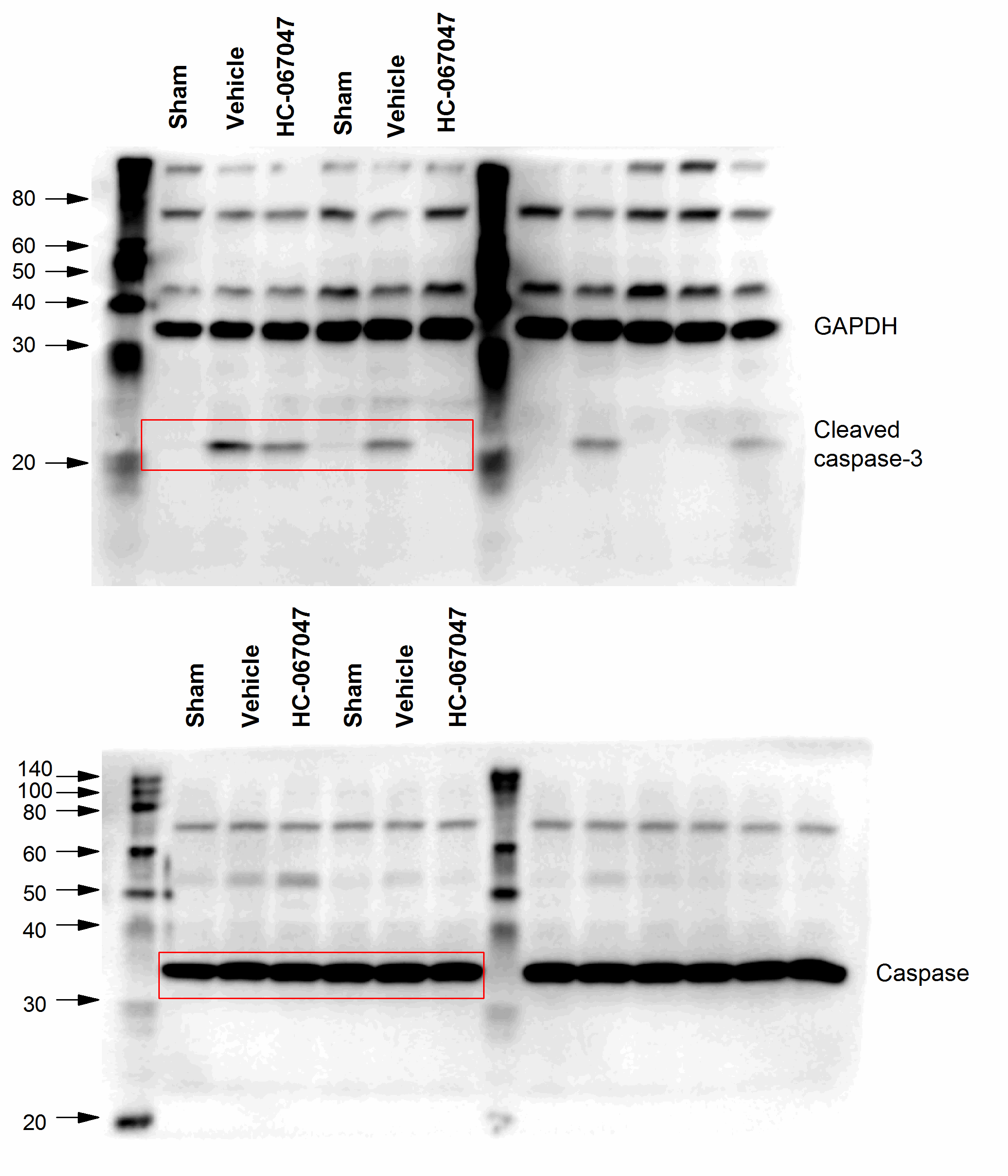
**

**Supplementary Figure 7 . The full-length blots/gels is the display of cropped gels and blots from Figure 5E.**

**
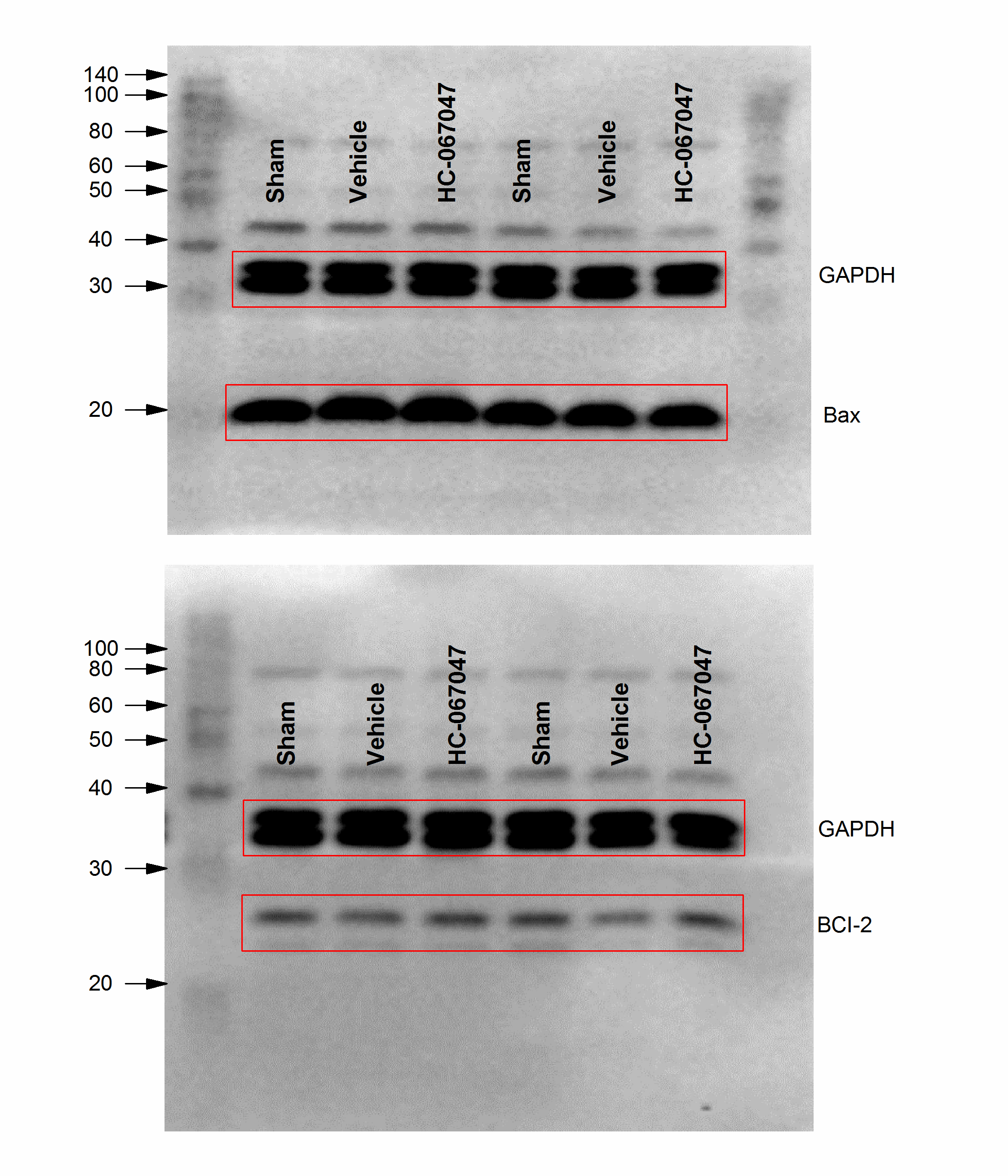
**

**Supplementary Figure 8 . The full-length blots/gels is the display of cropped gels and blots of P-AKT and AKT from Figure 7A.**

**
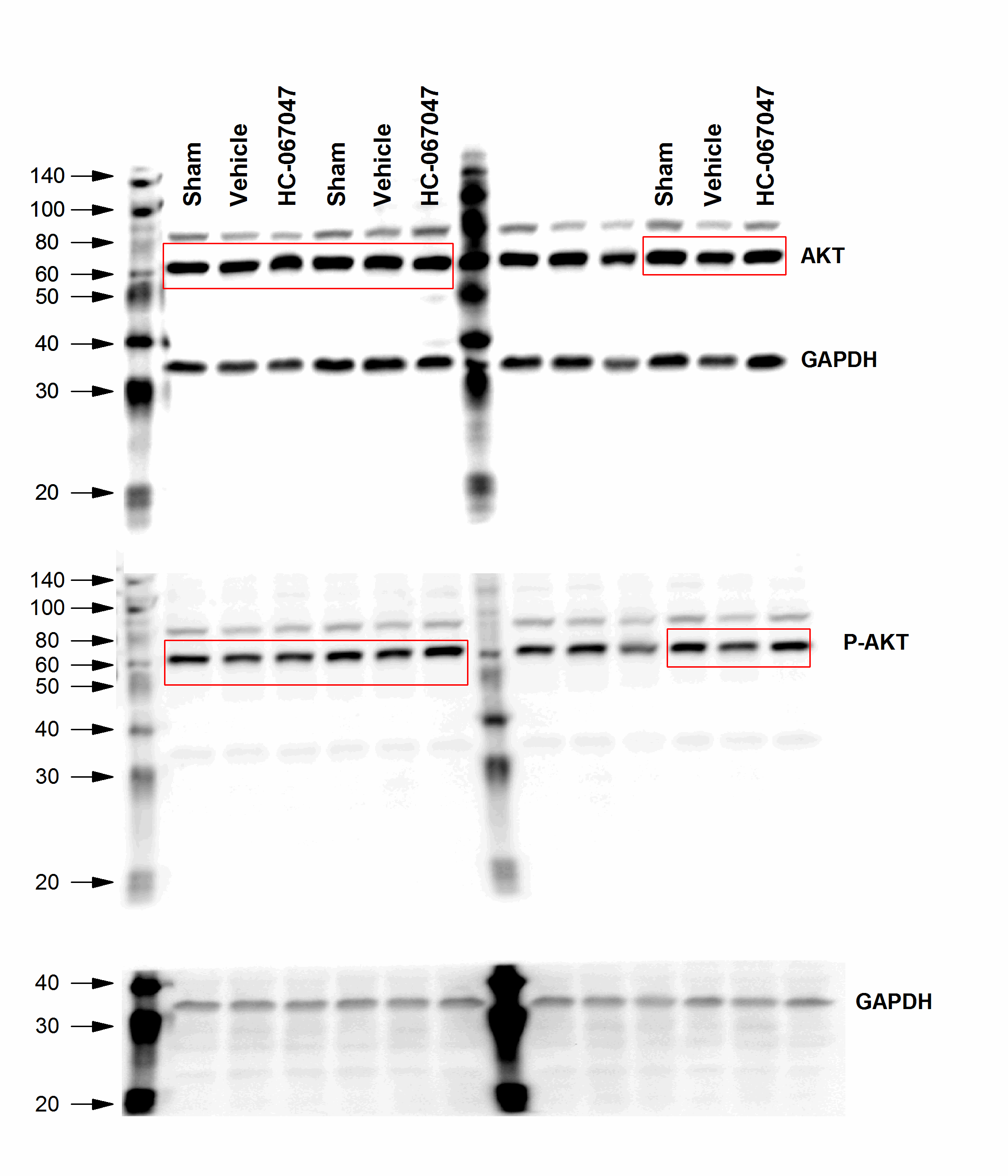
**

**Supplementary Figure 9 . The full-length blots/gels is the display of cropped gels and blots of P-ERK and ERK from Figure 7A.**

**
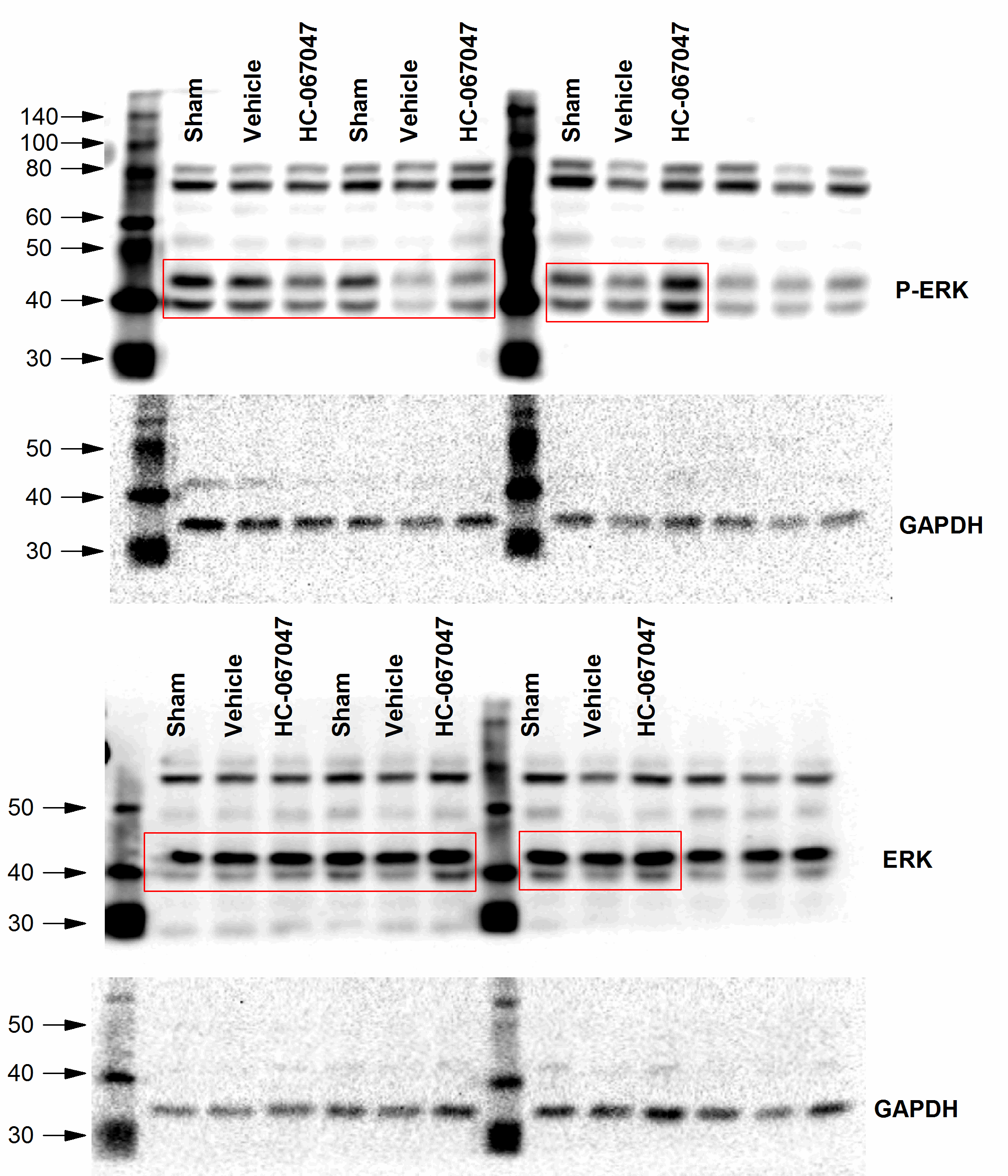
**

**Supplementary Figure 10 . The full-length blots/gels is the display of cropped gels and blots of P-GSK-3β and GSK-3β from Figure 7A.**

**
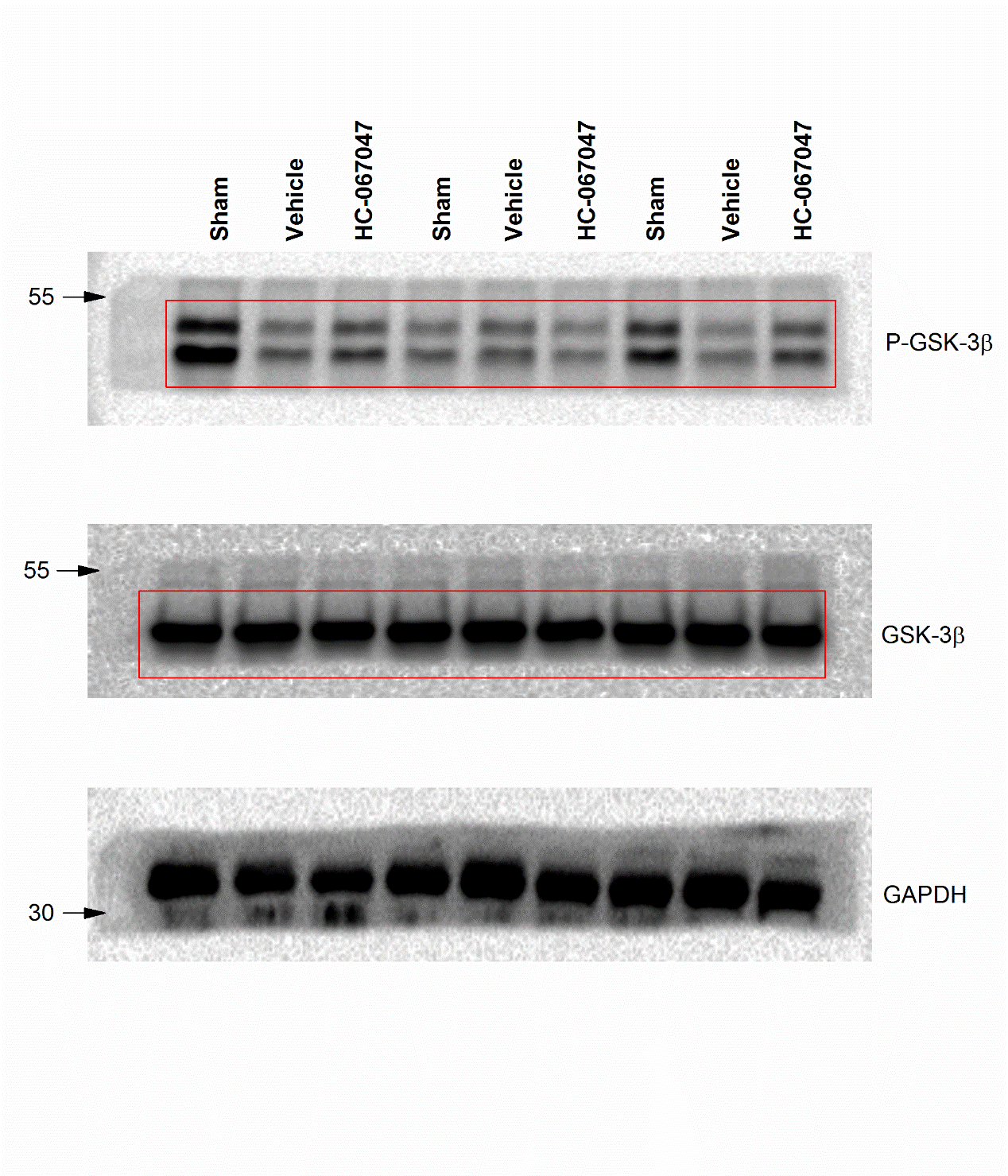
**

**Supplementary Figure 11 . The full-length blots/gels is the display of cropped gels and blots of P-STAT3 and STAT3 from Figure 7A.**

**
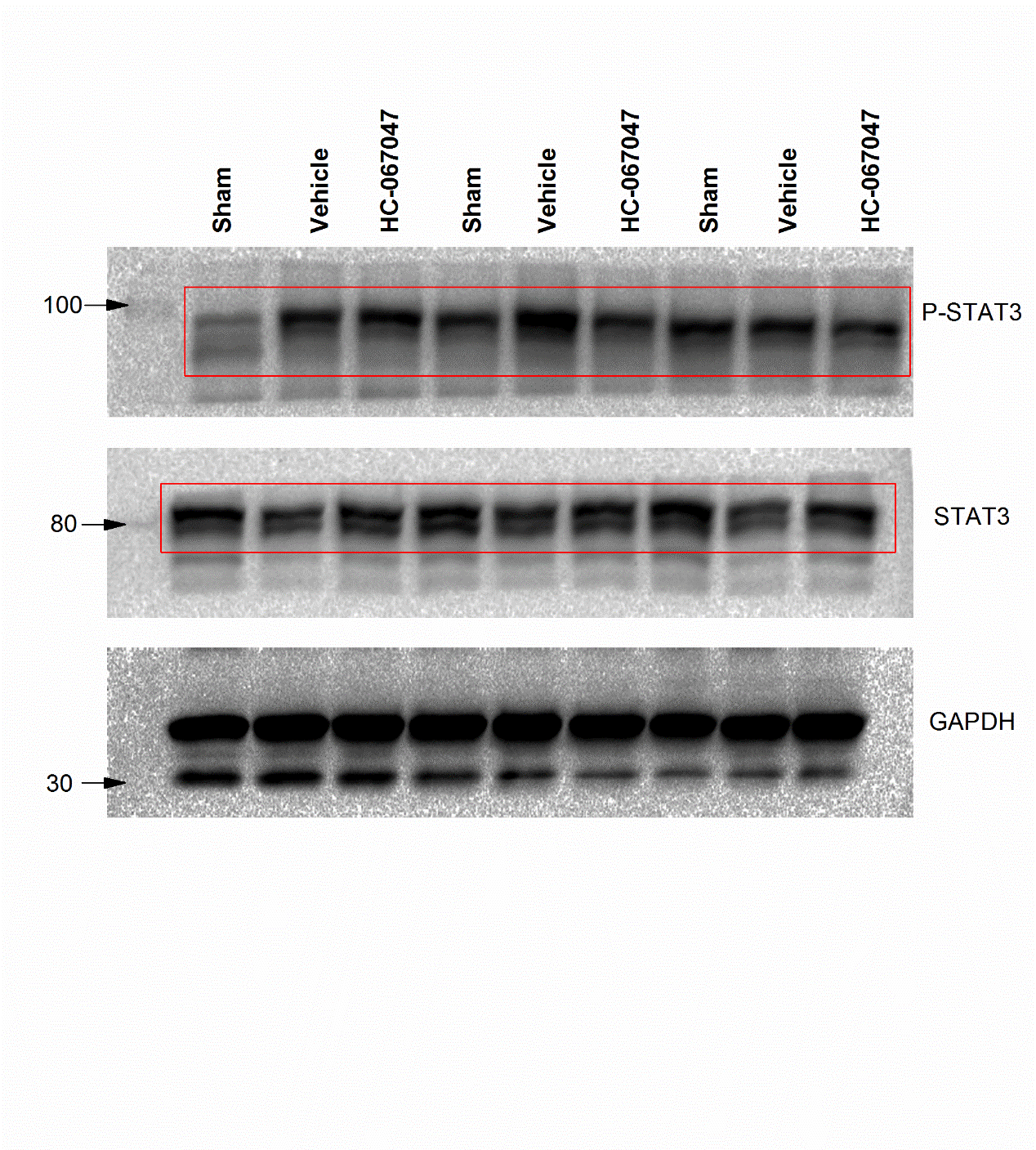
**
